# Manipulation of Gaseous Ions with Acoustic Fields at Atmospheric Pressure

**DOI:** 10.1021/jacs.4c01224

**Published:** 2024-05-08

**Authors:** Yi You, Julia L. Danischewski, Brian T. Molnar, Jens Riedel, Jacob T. Shelley

**Affiliations:** † Department of Chemistry and Biochemistry, Kent State University, Kent, Ohio 44242, United States; ‡ Division of Instrumental Analytics (1.3), Federal Institute for Materials Research and Testing (BAM), Berlin D-12489, Germany; § Department of Chemistry and Chemical Biology, Rensselaer Polytechnic Institute, Troy, New York 12180, United States

## Abstract

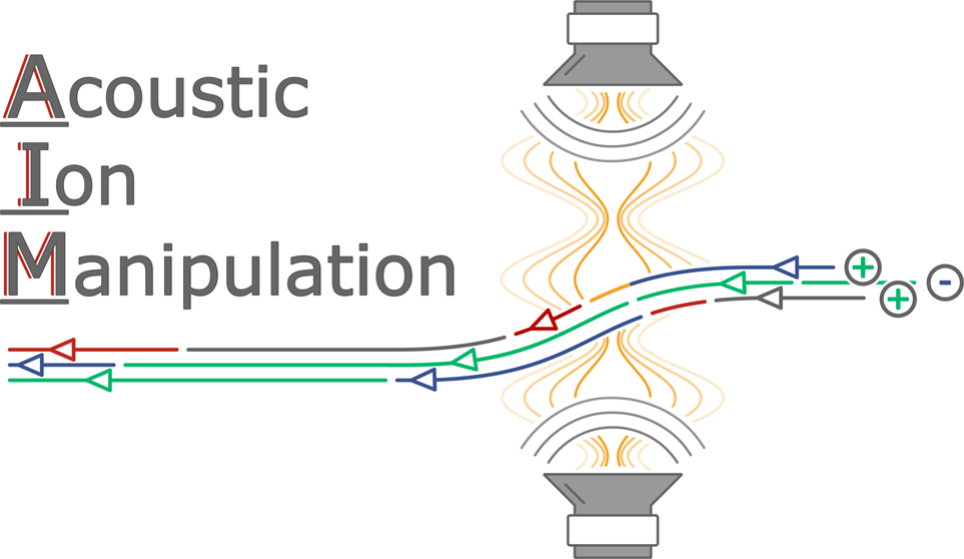

The ability to controllably move gaseous ions is an essential aspect of ion-based spectrometry (*e.g.*, mass spectrometry and ion mobility spectrometry) as well as materials processing. At higher pressures, ion motion is largely governed by diffusion and multiple collisions with neutral gas molecules. Thus, high-pressure ion optics based on electrostatics require large fields, radio frequency drives, complicated geometries, and/or partially transmissive grids that become contaminated. Here, we demonstrate that low-power standing acoustic waves can be used to guide, block, focus, and separate beams of ions akin to electrostatic ion optics. Ions preferentially travel through the static-pressure regions (“nodes”) while neutral gas does not appear to be impacted by the acoustic field structure and continues along a straight trajectory. This acoustic ion manipulation (AIM) approach has broad implications for ion manipulation techniques at high pressure, while expanding our fundamental understanding of the behavior of ions in gases.

## Introduction

Manipulation and control of the motion and direction of ionized particles and molecules have been pursued for more than a century.^1,2^ Because these species carry charges, most approaches utilize electromagnetic interactions to align, orient, or deflect dipoles and their trajectories via the electrostatic and/or Lorentz forces imposed by external electric and magnetic fields, respectively.^3^ The ability to carefully detect the responses of charged species within these well-controlled external force fields has led to a myriad of instrumental and technological developments toward analytical and industrial applications.^4,5^ From an analytical perspective, the electromagnetic potentials result in predictable and distinctive spatial and temporal dispersion of ionic species, which yields ion-specific information that can be translated into pertinent chemical insights. Based on these discoveries, methods such as electrophoresis, mass spectrometry (MS), and ion mobility spectrometry (IMS) have become cornerstones in contemporary sample purification and chemical analyses.^6,7^

While targeted guiding of ions, such as precise ion-trajectory control and ion trapping, in low-pressure environments are well established,^8^ many attempts have been made to achieve similar performance at atmospheric pressure.^9,10^ In comparison with high-vacuum conditions, the increased frequency of collisions under atmospheric conditions (*i.e.* 10^10^ s^–1^ at 1 bar) governs ion directionality, leading to the need for higher field strengths to manipulate (*i.e.* focus, deflect, gate, and separate) ions at elevated pressure.^3,11^ For instance, voltages ranging from hundreds of volts to several tens of kilovolts are commonly needed to overcome dominating aerodynamic and diffusion effects, which can translate to over 1000 V/m.^9,12^ At 1 bar of N_2_, a singly charged ion would gain only *ca.* 7 × 10^–5^ eV on average between collisions at this field strength, which is far below thermalized ion energies (*i.e.* 4 × 10^–2^ eV at 298 K). As a result, ion diffusion within these fairly large electric fields is quite significant. With decreased pressures of 1 mbar N_2_, electrostatic ion optics are more efficient because collisional frequency drops to 10^7^ s^–1^ while the average energy gained between collisions of *ca.* 7 × 10^–2^ eV exceeds thermalized energy. The consequences of frequent collisions between a target molecular ion and the surrounding species in the medium diminish the intended effect of manipulation methods with electrostatic and magnetic fields.^13−15^ A more elegant way to manipulate the ion behavior in a collision-governed system is to directly leverage the charge dependence of the collisional cross sections. Collision-controlled dynamics in continuous media, thus far, are most commonly used in mobility-focused applications, which aim to achieve retardations in speed based on the interaction cross section between ions and neutral gas. From this perspective, developments in IMS incorporate electrodynamic, magnetic, and gas-dynamic (field-flow) approaches, which have led to advances in ion funnels^16,17^ and a vortex-stream ion guide.^18^ However, methods that exploit bulk media interactions (*e.g.,* acoustics) to enable controlled trajectories of a traveling ensemble certainly exist and have been gaining increased attention in recent years.^19^ This specific class of methods is commonly referred to as acoustic levitation^20−23^ and acoustic tweezers.^24,25^ These use a rapidly alternating acoustic pressure gradient that effectively reaches a dynamic equilibrium and exhibits static properties. The result is that a target object can be moved and controlled on a much longer time scale (*e.g.*, seconds to minutes) compared to that of a periodic acoustic field (*e.g.*, micro- to milliseconds).

Noncontact object handling and spatial confinement of objects have enabled discoveries in many fields.^22,26−29^ The appeal of these methods, particularly from a chemical perspective, lies in their abilities to avoid surfaces, making noncontacting approaches attractive for synthesis, analytical purposes, and beyond.^26,30,31^ Especially, aligned with the concept of direct mass-spectrometric analyses for their simplicity,^29,32−34^ the missing puzzle piece seems to be the combination of these two into a versatile sampling platform. Plasma-based ionization sources for MS certainly qualify as ideal candidates, where reagent ions produced by an electrical discharge allow highly efficient chemical detection and quantification; examples include direct analysis in real time (DART), flowing atmospheric-pressure afterglow (FAPA), and low-temperature plasma (LTP) probe.^35−39^ Conceptually, analytes in condensed-phase samples held within an acoustic levitator could be desorbed, ionized by an ion source, and transported to a mass spectrometer. However, during our attempts to achieve this goal with an analyte-containing levitated droplet, no analyte signal from the species within the droplet was detected. In fact, ions that were produced by the source and carried by the thin gas stream (*e.g.*, ∼2 mm diameter at 3–5 m/s linear velocity) were not detected at all when using an acoustic levitator as the droplet sample holder. At this point, we noticed that the presence of a resonant acoustic field might have obstructed the ion beam from entering the atmospheric-pressure inlet of the mass spectrometer. In other words, the acoustic force field altered the ion beam trajectory, even if the ions were carried and shielded by a neutral stream of gas. The inability to detect ions downstream indicates that acoustic levitation may be unsuitable for sample introduction in direct mass-spectrometric analysis. However, this observation offers intriguing insight into the interaction between the acoustic field and ionic species. Specifically, it suggests that, alongside electric and magnetic fields, acoustic fields could serve as an additional means to manipulate beams of charged species.

This work describes the behavior of small ions (*e.g.*, less than 100 u) within a neutral gas beam as they traverse through resonant acoustic field structures. These ions preferentially migrate toward the regions of static pressure, while the neutral gas maintains its initial trajectory. This acoustic ion manipulation (AIM) approach, as we refer to it, is shown to be capable of the four major uses of electrostatic and magnetic ion optics: (i) deflection, (ii) gating, (iii) focusing, and (iv) fractionation/separation. Importantly, the AIM phenomenon achieves this with only acoustic fields and does not introduce physical obstructions that would otherwise lead to ion losses and contamination over time. As such, the AIM technique could have broad utility in any area that relies on ion processing at or near atmospheric pressure, including ion mobility and mass spectrometry, materials processing, and chemical synthesis.

## Results and Discussion

**Figure 1:**
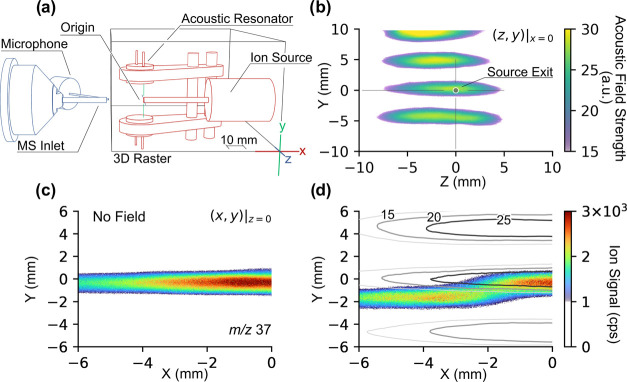
Experimental mapping of ion deflection within an ultrasonic resonator. (a) Diagram of the setup used for ion beam mapping. The components outlined in blue and red represent static and moving parts, respectively. The relative position between the ion source and the acoustic resonator remained constant during mapping. (b) Acoustic field strength at *x* = 0 in arbitrary units. The acoustic sensing circuit used is depicted in Figure S3b. The ion paths for (H_2_O)_2_·H^+^ at *m*/*z* 37 in the absence (c) and presence (d) of the acoustic field within the resonator are shown in their respective panels. (d) Simultaneously measured acoustic pressure field is superimposed with the ion trajectory. The origin, (*x*, *y*, *z*), is defined by the outlet of the ion source, where (*x* = 0, *z* = 0) is defined on the center line aligned with the outlet and the (*y* = 0) is defined at a point 5 mm away from the ion exit.

Because it is merely impossible for the source-produced ions to be fully depleted by only traveling through a resonant acoustic field in the open air, ion distributions measured by MS and the acoustic pressure field were mapped simultaneously in three-dimensional space to determine ion paths as they traverse through an acoustic pressure gradient (Figure 1a and Section S1). To investigate the ion-acoustic interaction more accurately, in this scenario, we used nitrogen as the discharge gas at 0.85 L min^–1^ and allowed the ion-containing gas to be realized from a cylindrical tube from the source in a controlled and laminar manner. Meanwhile, a corona discharge sustained at 5 kV, which required ∼0.67 μA of current, was used to minimize heat production to avoid thermally related acoustic-medium interactions.

Without the presence of the acoustic field, the ion trajectory was nearly straight toward the mass spectrometer inlet (Figure 1c), which is expected, given the laminar gas flow exiting the source. With (H_2_O)_2_·H^+^ at *m*/*z* 37 as an example, the presence of a standing acoustic wave immediately deflected the ions away from their original trajectory (Figure 1d). The superimposed acoustic field contour and the deflected ion trajectory indicate that the ions followed the acoustic pressure gradient, traveling from an antinode toward the adjacent static-pressure node. A similar effect was also observed for O_2_^+^ at *m*/*z* 32 (Figure S4). Notably, the acoustic field strength was quite weak. In this example, where ∼30 V_p–p_ was used, the resonator was only capable of levitating a ∼2 mm diameter polystyrene foam bead with a mass of ∼50 μg or ∼5% of water with equivalent volume. Levitating a water droplet would require a much higher voltage/power input on the piezo transducers (∼120 V_p–p_). This finding indicates that ions or an ion-containing stream are more sensitive to acoustic forces than larger (*i.e.* comparable to the acoustic wavelength) neutral objects. Compared to object levitation, ion deflection with resonant acoustic fields requires considerably lower acoustic power.

The stunning observation of acoustically induced ion deflection immediately raises questions regarding the underlying mechanisms and processes. One aspect that can be immediately ruled out is the possibility of an electric field inside the acoustic resonator. In the deflection experiment shown above, the voltage on the transducers was ∼30 V_p–p_, which translates to a maximum possible electric field strength of 1.6 V/mm; this is several orders of magnitude lower than that used in electric-field-based ion deflectors, despite the voltage drop on the transducer by sound emissions. Additionally, the speakers have an electrically grounded case and screen over the transducers; therefore, no electric field was present in the resonator volume.

**Figure 2:**
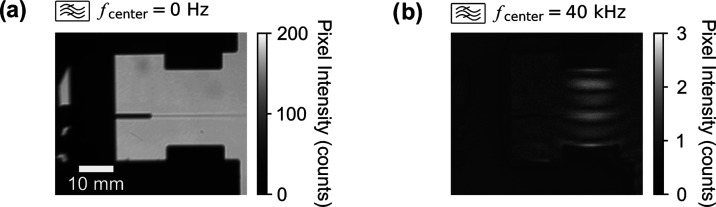
Direct observation, using the defocusing shadowgraphy method, of a partially ionized N_2_ gas stream containing a trace amount of isopropanol (as a contrast agent) traveling through an antinode (bright regions in (b)) within the acoustic resonator. The gas flow rate and transducer voltages were 0.85 L min^–1^ and 30 V_p–p_, respectively, which are the same as in the ion-deflection experiment. A corona discharge was used here with the discharge voltage and current at 5 kV and 270 μA, respectively. The stroboscopic triggering of the light source enabled the camera to capture the transient acoustic field; different frequency components can be subsequently isolated with bandpass filters. More detailed information can be found in the Section S3.

The other possibility involves the deflection of most of the neutral gas stream, which serves as the carrier for the ions. This hypothesis has been ruled out by independently mapping the neutral gas and ion profiles as they traverse the resonant acoustic field structure. A trace amount of isopropanol vapor was added to the N_2_ source gas as a contrast agent, which allows direct visualization of the gas stream traveling through the acoustic field with stroboscopic defocusing shadowgraphy (see Section S3).^40^ Here, the back illumination was also pulsed with a 0.4 Hz frequency difference from the piezo driving frequency. Effectively, the images that were recorded at 4 frames per second can be used for time-domain Fourier transform, from which the gas stream and transient acoustic field can be measured simultaneously. Based on this result, it is possible to deduce that the presence of an acoustic field did not appreciably affect the neutral gas stream, which contains ions produced from a corona discharge while traveling through the antinode (*cf.*Figure 2a). In contrast, the same acoustic field was sufficient to deflect the ion beam by ∼2 mm. Due to the low ion density, it is not possible to optically observe the ion flow path itself. Nonetheless, it is possible to conclude that the ion stream was split from at least the vast majority of the neutral gas and formed a separate stream through the node region. As additional confirmation, shadowgraphy images of an entirely neutral gas stream (*i.e.* without a corona discharge) showed no deflection by the antinode of the acoustic resonator (*cf.*Figure S8) under these conditions. In fact, the images in Figures 2 and S8 are nearly identical.

One possible hypothesis for the cause of acoustic ion manipulation is the additional long-range interaction force due to the presence of ions. More specifically, the effective distance of electrostatic or Coulombic forces is significantly greater than that of van der Waals forces (*e.g.*, London dispersion, Keesom and Debye forces). Consequently, the additional Coulombic interactions increased the effective collision cross section, resulting in increments in the compressibility coefficient of an ion-containing gas stream, which can be reflected by the change of internal energy versus volume, or ∂*U*/∂*V*. This proposed mechanism still requires further experimental investigation, which will be the focus of a later study. However, a similar acoustic-induced fractionation within aqueous media has been reported.^41,42^

**Figure 3:**
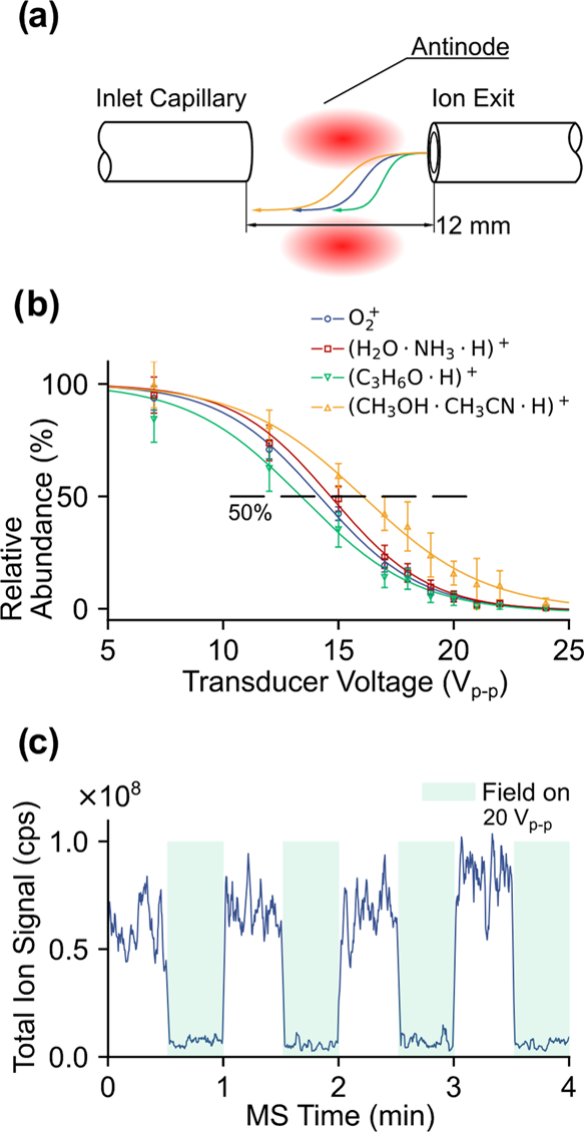
Ion fractionation and gating with resonant acoustic fields. (a) Schematic of gating mode. Ion-specific response curves with acoustic field strength, shown as ultrasonic transducer voltage, are shown in panel (b). The solid markers are experimentally measured data, while the solid traces are sigmoidal fits to these data. Panel (c) shows the basic ion shutter operation at a fixed transducer voltage, 20 V_p–p_. The term “cps” stands for “counts per second”.

Thus far, we have demonstrated that the presence of an acoustic field can deflect ions by redirecting trajectories. To gain a more complete picture, the acoustic pressure was increased by changing the transducer voltages to reveal acoustic ion interactions. Specifically, the outlet of the ion source and the inlet of the mass spectrometer were aligned with each other, maximizing the ion transmission and detection in the absence of an acoustic field (*cf.*Figure 3a). In this “gating geometry,” the characteristic loss of ion signal with increasing transducer voltage exhibited a clear dependency on ion identity, *e.g.*, *m*/*z* and/or collision cross section (cf. Figure 3b). Figure 3b shows the gating response for O_2_^+^, (H_2_O·NH_3_·H)^+^, protonated acetone (C_3_H_6_O·H)^+^, and pronated methanol acetonitrile cluster (CH_3_OH·CH_3_CN·H)^+^ at *m*/*z* 32, 36, 59, and 74, respectively. Importantly, these ions were selected because they have different chemical origins. One feature is that protonated acetone at *m*/*z* 59 was deflected at a weaker acoustic field compared to the other species. It is possible that the permanent dipole associated with the carbonyl group on acetone results in stronger intermolecular interactions compared to other ambient species, which could be reflected in gas compressibility that can be further translated into a greater ion-acoustic sensitivity. Practically, these results indicate that a resonant acoustic field could be used as an ion separator, similar to a differential mobility analyzer. From the ion-acoustic interaction, we term this method acoustic ion manipulation (AIM).

Notably, the AIM approach operates outside the mass spectrometer in the open air. That is, AIM preselects ion clusters before entering the reduced-pressure environment of the mass spectrometer and undergoing collision-induced dissociation. More specifically, ions that stem from the same chemical origin exhibit similar or identical ion-acoustic responses (*e.g.*, NH_3_·H^+^, H_2_O·NH_3_·H^+^, and (H_2_O)_2_·H^+^ at *m*/*z* 18, 36, and 37, respectively, Figure S9). At this current stage, it is not possible to correlate the deflection of specific *m*/*z* values with the transducer voltages for the ions detected here. However, this does not exclude that possibility for more massive molecular ions, where the analytes would be much larger than the solvent/matrix clusters observed with the current plasma sources. This initial investigation focuses on the AIM phenomenon for small molecular ions and clusters. Although larger ions are perhaps of greater relevance for modern applications, their use also presents a host of several additional factors (*e.g*., more extensive solvation shells, a variety of conformations, large solvent droplets) that obfuscate the ability to track the motion of molecular-sized charged particles within these acoustic environments. In follow up studies, we will explore the behavior of larger (*e.g.*, biomolecular) ions in AIM.

**Figure 4:**
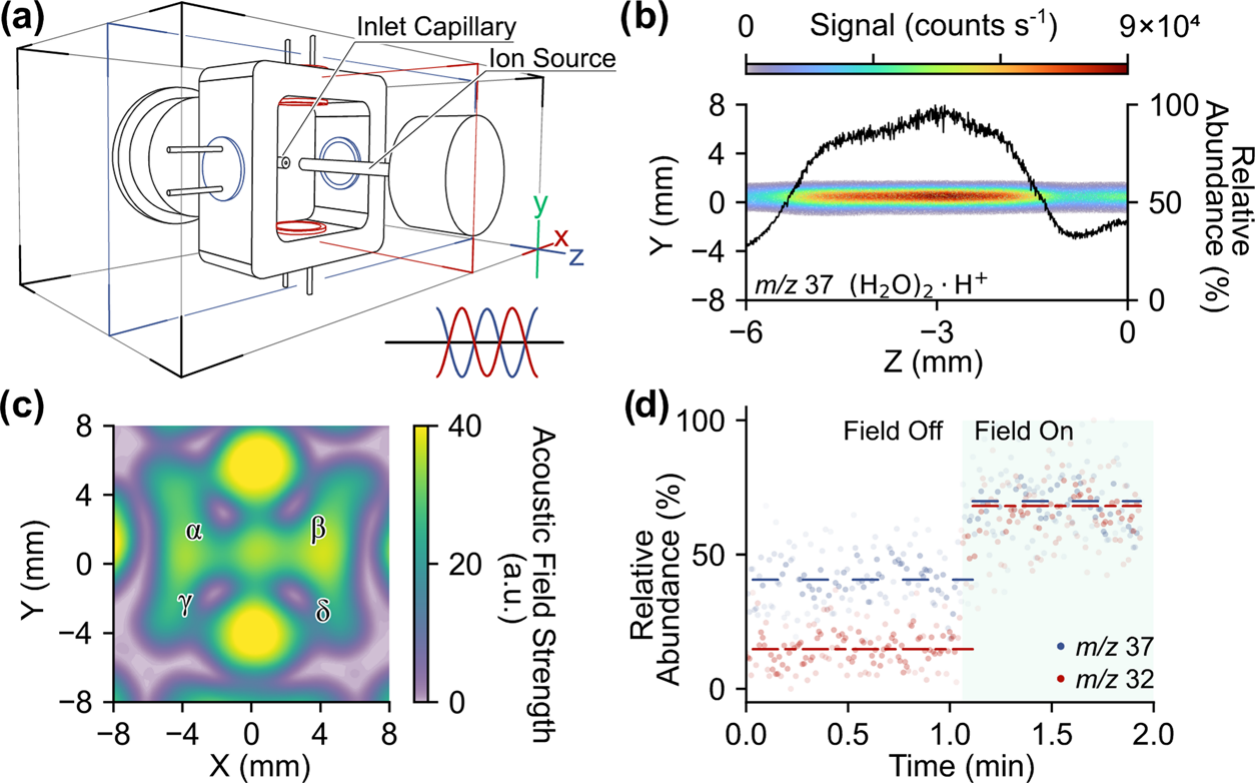
Two-dimensional acoustic resonator array for pseudo ion focusing. A simplified schematic is given in panel (a). The dimensional drawing is given in Figure S10. In this configuration, the two pairs of transducers are color-coded in red and blue, denoting their driving polarity. The *y*–*z* section view, given by the red plane in panel (a), of the (H_2_O)_2_·H^+^ ion at *m*/*z* 37 is shown in panel (b). The solid-black trace shows the vertically averaged ion abundance (right axis). Panel (c) shows the center slice, denoted by the blue plane in panel (a), of the acoustic field. Panel (d) represents the ion abundances of O_2_^+^ ion at *m*/*z* 32 and water cluster at *m*/*z* 37 at the “focal point” with and without the acoustic field. The dashed lines in red and blue represent the average ion abundance with respect to the presence of the acoustic field.

While the above results utilized a one-dimensional standing acoustic wave for ion manipulation, more complex two- and three-dimensional acoustic patterns could be used to enhance the ion-optic capabilities of AIM. This includes gating, focusing, deflection, and ion fractionation. One example that has been explored is the field structure shown in Figure 4c, which was achieved using four ultrasonic transducers in two pairs in differential phase. In the experiment, the outlet of the ion source was aligned with one of the node channels (point α in Figure 4c). The spatial distribution of the (H_2_O)_2_·H^+^ ion at *m*/*z* 37 was then raster-scanned in three dimensions. Compared to the monotonic ion decay observed without the acoustic field (Figure 1c), the presence of the resonant acoustic field generated a “hot spot” where the ion abundance was significantly higher than in other regions. Notably, the four nodes in this structure (*cf.*Figure 4c) were basically the same size and shape. As such, when an ion source was pointed at each, they all exhibited the same degree of focusing ability and an increase in ion signal. Here, we arbitrarily chose node-α as an example to demonstrate ion focusing with a 2D AIM.

To evaluate the impact of pseudo ion focusing, a semiquantitative approach was employed by positioning the mass spectrometer inlet at the center of the hot spot. By toggling the acoustic field, the relative abundances of the two example ions, O_2_^+^ ion at *m*/*z* 32 and (H_2_O)_2_·H^+^ ion at *m*/*z* 37, showed a significant increase by 3.6- and 1.7-fold, respectively. It is important to note that the increase in the abundances corresponded to the characteristics of focusing; however, the absence of a beam waist and other structural attributes certainly indicate the need for further investigation into the design of specific acoustic fields.

## Conclusions

This proof-of-concept demonstration highlights the potential of the AIM concept to manipulate ions and ion clusters in the open air, yielding a low-power (<1 W) and flexible atmospheric pressure ion optic. This obviates the need for high voltages or magnetic fields to focus or guide ions at high pressures. The AIM concept allows ion gating, deflection/redirection, focusing, and fractionation. Unlike most ion optics, AIM does not introduce physical obstructions or become contaminated during use. These attributes should yield higher transmission and sensitivity with minimal maintenance. At the very least, AIM platforms can be used to replace, supplement, or complement existing high-pressure ion optics and ion mobility analyzers, but in a more flexible manner. More specifically, a simple acoustic differential mobility analyzer could be achieved by ramping the transducer voltage to flag the ions or ion clusters, reflecting their source or even identities. By leveraging the wave-like properties of sound and recent advancements in phased array ultrasonics, complex acoustic structures can be easily generated on demand. It is easy to envision the possibility of making time-dependent pressure fields that resemble those of electrical quadrupoles and, perhaps, offer similar ion-optic functionality. Additionally, the known acoustic-particle interaction may allow manipulation of both bare and solvated ions simultaneously or differentially, which could extend the functions of spray sources, such as electrospray and nanospray, for chemical analysis and synthesis (*e.g*., electrospinning). Based on the observations in this work, AIM shows potential to serve as a versatile ion guide that is adaptable in real time and on-site.

## Data Availability

Data from this work is available upon request. Code used for image rendering can be found in the Supporting Information.

## Supporting Information Available

Section S1: Experimental. Ion source (Figure S1); typical mass spectrum (Figure S2); and ultrasonic driving and sensing circuits (Figure S3). Section S2: Deflection of other ions. Deflection of O_2_^+^ ion (Figure S4) and cross-validation with an ionCCD (Figure S5). Section S3: Optical characterization of the linear acoustic resonator. Introduction to the defocusing shadowgraphy system (Figure S6); stroboscopic image series processing (Figure S7); and image of a neutral stream traveling through the acoustic field (Figure S8). Section S4: AIM ion cluster fractionation; acoustic ion response curve for water/ammonia clusters (Figure S9); and deflection mode operation of AIM (Figure S10). Section S5: Appendix. Dimensional drawing of the 2D acoustic resonator (Figure S11) (PDF)
