# Supplementary material for: Manipulation of Gaseous Ions with Acoustic Fields at Atmospheric Pressure
Source: J Am Chem Soc. 2024 May 8;146(21):14587–92. doi: 10.1021/jacs.4c01224 (PMC11140733; doi:10.1021/jacs.4c01224)
Supplement: Supplementary file 1 — ja4c01224_si_001.pdf [file ja4c01224_si_001.pdf]

Supporting information for:

Manipulation of Gaseous Ions with Acoustic Fields at Atmospheric Pressure

Yi You[1, 2], Julia L. Danischewski[3], Brian T. Molnar[3], Jens Riedel[2*], and Jacob T. Shelley[1, 3*]
[1] Department of Chemistry and Biochemistry, Kent State University, Kent, OH 44242, USA
[2] Division of Instrumental Analytics (1.3), Federal Institute for Materials Research and Testing (BAM), Berlin, D-12489, Germany
[3] Department of Chemistry and Chemical Biology, Rensselaer Polytechnic Institute, Troy, NY 12180, USA

[*] Corresponding Authors: Jacob T. Shelley, Email: shellj@rpi.edu
Jens Riedel, Email: jens.riedel@bam.de

Table of Contents

1. Experimental design and details....2
2. Deflection of other ions 5
3. Optical characterization of the linear acoustic resonator....7
4. AIM ion cluster fractionation 10
5. Appendix....12
7. References....14

1. Experimental

In this section, the experimental design details will be provided from two perspectives, which are sourcing and sensing. Sourcing refers to the ion generation or formation of the ion beams in specific, as well as the generation of resonant acoustic fields. Sensing involves the measurements of ion current, ion specification and distribution, and acoustic pressure field. Importantly, all experiments included in this work were performed in an open laboratory environment.

Ionization source: In this study, we used an enclosed chamber to generate ion beams consisting of different ionic species. Specifically, we employed the pin-to-capillary flowing atmospheric-pressure afterglow (FAPA) geometry. For simplicity, we will refer to these ion sources as FAPA throughout the study (*1*). Unlike the typical FAPA operating conditions that involve a high-current, low-voltage glow discharge mode, we exclusively used the corona discharge mode to minimize excessive heat generation. To accommodate the different experiments conducted in this research, we employed multiple FAPA sources with slightly varied geometry and operating conditions. These specifications will be provided in the associated contexts. In general, the discharge gas entering the chamber was regulated by either a mass flow controller (e.g., GR116-A-PS, 5 SLPM, Fathoms Technology) or a rotameter. It is important to note that either the mass flow controller or the rotameter was calibrated prior to experiments using a standard gas flow calibrator (Gilibrator-2 Calibrator, Gillian, Sensidyne LP).

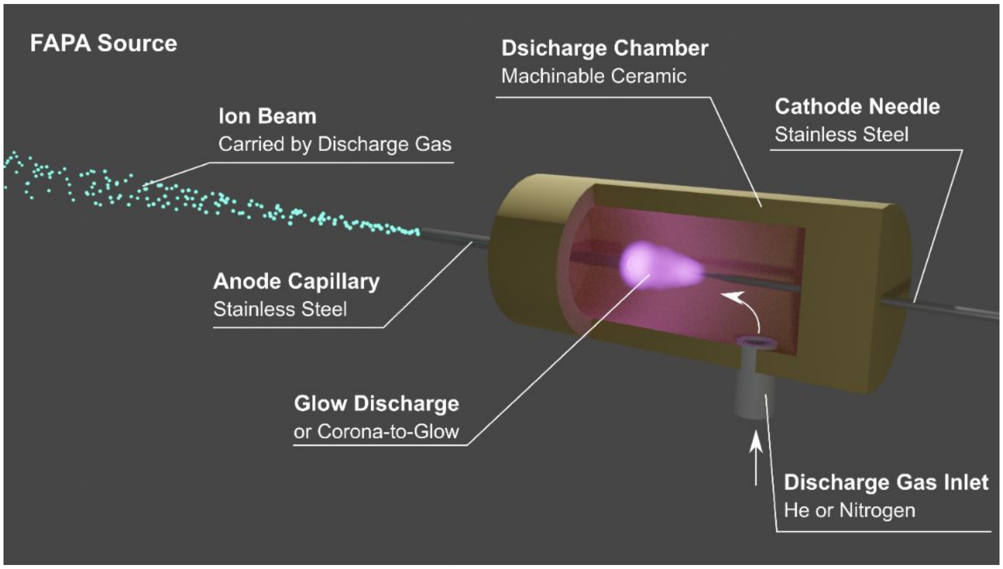

Figure S1. A 3D presentation of a typical FAPA source.

Mass Spectrometry: A unit-resolution linear ion trap mass spectrometer (LTQ-XL, Thermo Scientific, San Jose, CA) was used to record low-mass ion signals. The mass range was set to *m/z* 20-200 with the injection RF low-mass cutoff of *m/z* 15, which enable detection of ions down to *m/z* 20. The capillary temperature and maximum injection time were set to 275°C and 20 ms, respectively. Because the reagent ions were the primary targets, no further tuning was performed on the instrument. With the mass spectrometer configuration set as described above, a FAPA source was operated under current-limiting mode at 4.94 kV and 0.67 µA. Fig. S2 shows the mass spectrum when nitrogen was used as the discharge gas at 0.40 L min^{-1}.

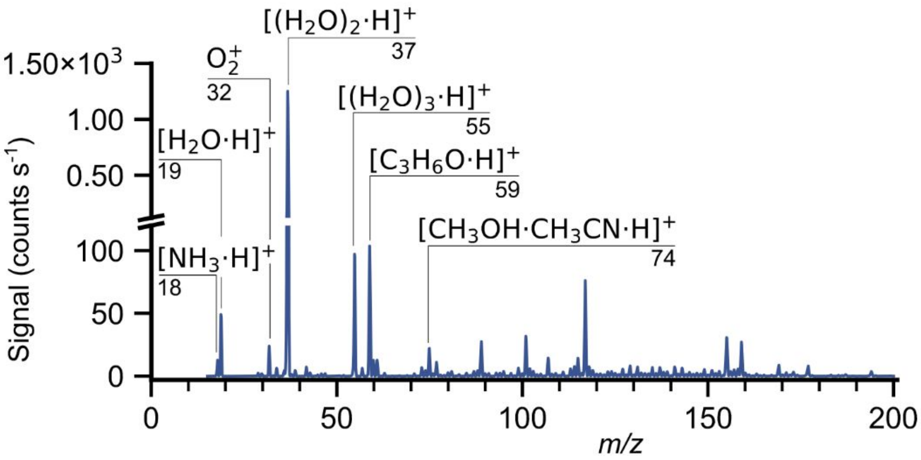

Figure S2. Typical background mass spectrum from a nitrogen-based FAPA source exposed to the open laboratory environment recorded on an LTQ-XL mass spectrometer. Note that the observed methanol and acetonitrile ions are due to residual solvent vapor in the laboratory air.

Ion Detector Array: To cross validate the ion deflection and avoid the possible interferences from the pull of the vacuum of the mass spectrometer interface, an ion-sensitive charge-coupled device (IDS-2030 ionCCD, Xylem, Birmingham, AL) was also used.

Ultrasonic Speaker Driver: An ultrasonic driver circuit was designed and built in-house to power the ultrasonic speakers (TCT40-16, Waitrony, Hongkong, China) in the present study (Fig. S3a). Most commonly, the ultrasonic speakers can be directly driven by function generators, as they are rated for 10 $V_{p\text{-}p}$. However, a customized circuit was more flexible in terms of voltage range and improved electric-sound energy conversion efficiency, which are highly desirable for the present experiments. Therefore, a circuit based on the resonant reset forward topology was meticulously adjusted to meet the requirements of the experiment (*2*). In specific, the circuit utilized a pulse-width modulation (PWM) controller integrated circuit (TL494, Texas Instrument, TX) to generate the main clock signal at a frequency of 40 kHz. To ensure stable operation and prevent frequency drifting, a separate gate driver integrated circuit (MC34151, Texas Instrument, TX) was used to buffer the PWM signal, minimizing thermal accumulation on the clock source.

On the power side, a homemade ferrite-core transformer (without air gap) was pumped by a power metal-oxide-semiconductor field-effect transistor (MOSFET, IRF540N, Infineon Tech., Berlin, Germany). The secondary side of this transformer was directly connected to the ultrasonic speakers through a matching inductor. In this study, several transducer configurations were used, which were one and two pairs. Notably, the ultrasonic speakers (transducers) within an acoustic resonator should be considered as one entity. In the resonators that were powered by this circuit, all ultrasonic speakers were connected to a common output terminal on the power supply, rather than being driven individually.

To regulate the voltage applied to the transducer(s) and control the travel distance of the piezo surface, which directly affects the output sound pressure, we implemented a monitoring network in the form of a half-bridge rectifier. This allowed us to adjust the voltage/power applied to the transducers. Furthermore, this monitoring network enabled resonance tuning. More specifically, the readout, V_{mon}, reaches maximum when the distance between a transducer pair is $n\lambda/2$, where n is a positive integer.

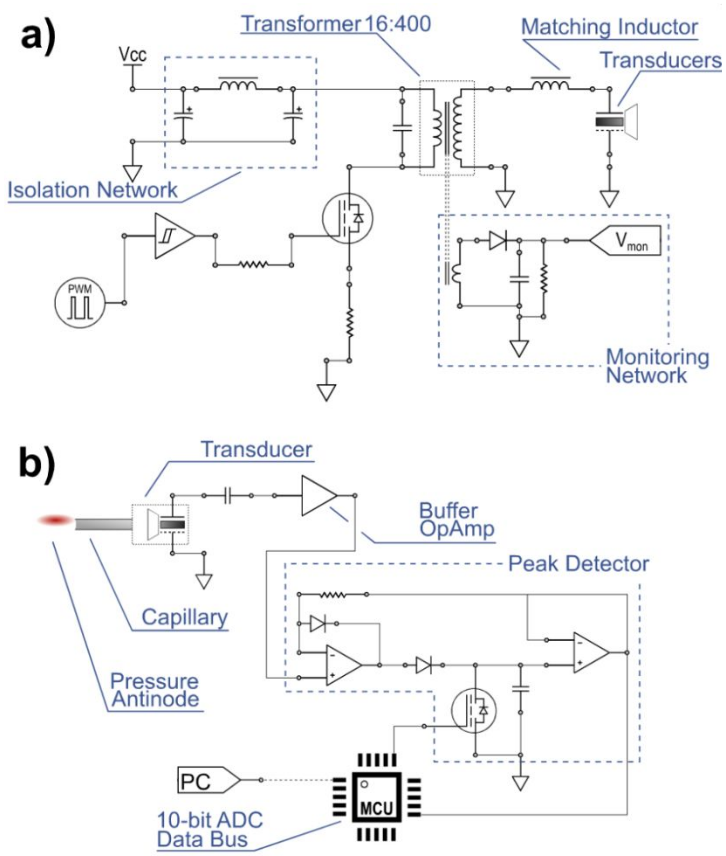

Figure S3. Simplified schematic of the ultrasonic driver circuit. The transducer driver circuit and acoustic field mapping circuit are given in panel (a) and (b), respectively.

Acoustic field mapping: Acoustic field strength mapping was conducted using an independent ultrasonic transducer (TCT40-16) as a microphone. To ensure spatial resolution, the transducer was enclosed in a 3D-printed case with a protruding capillary made of stainless steel (1.0 mm OD, 0.9 mm ID). This capillary was aligned with the mass spectrometer inlet capillary (Fig. 1a). Although the sensing assembly was not calibrated for absolute sound pressure determination, it was sufficient to measure the spatial distribution of the acoustic field. Relative acoustic field strengths were recorded using a peak detector (Fig. S3b). Specifically, the sensing transducer was capacitively coupled to a buffer operating amplifier (Op-Amp, LM324, Texas Instrument, TX), which had a gain of 100 to amplify the signal. Subsequently, the signal was passed through a peak detector circuit to determine the maximum amplitude. The final peak voltage was then digitized by the on-board analog-to-digital converter on a microcontroller (MCU, Atmel ATMEGA2560, Microchip Tech., AZ) with 10-bit resolution. Importantly, for each sampling point, the peak values were recorded and averaged 500 times to ensure statistical validity. To achieve this, the MCU sent a trigger signal to the MOSFET to discharge the accumulation capacitor in the peak detection loop after reading the peak voltages. The final digitized peak voltages were sent to a controlling computer, synchronized with the current (x, y, z) position of the motorized stage. The firmware on the MCU was programmed and compiled using Atmel Studio 7.0.

2. Deflection of other ions

Deflection of O_2^+ ions in an acoustic resonator

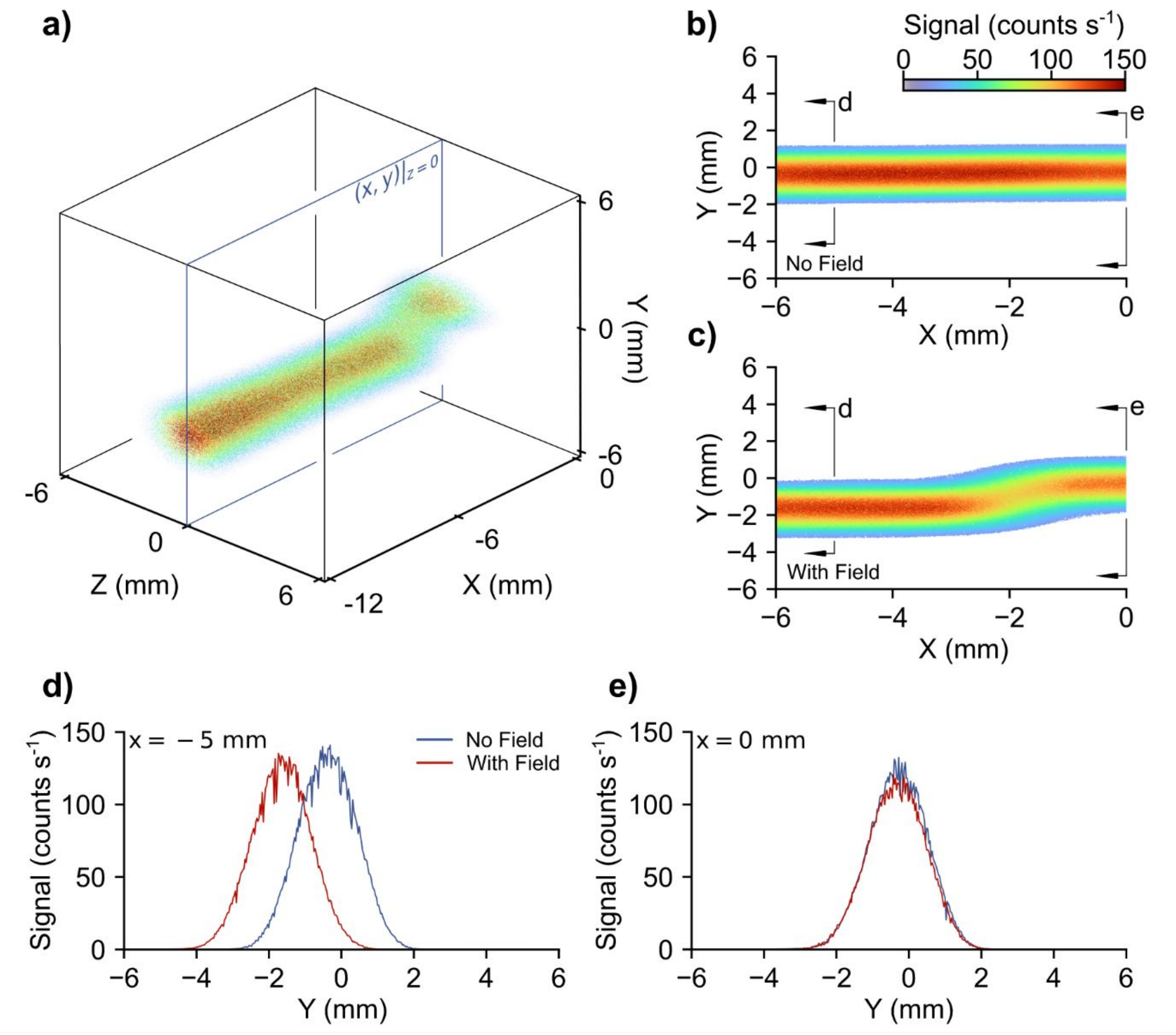

Figure S4. Experimental visualization of the deflection O_2^+ ion (*m/z* 32) within a resonant acoustic field. Panel (a) shows a 3D representation of the ion deflection. Panels (b) and (c) highlight the altered trajectory of O_2^+ ions when an acoustic field is applied. The color scales remain consistent across all three subfigures. Panel (d) and (e) provide a section view at x = -5 and 0 mm, after and before entering the acoustic field, respectively.

The acoustic response of O_2^+ ions (*m/z* 32) is illustrated in Fig. S4 along with the demonstration presented in the main text (Fig. 1). Similar to that of the *m/z* 37 ions, the trajectory of O_2^+ ions were deflected by ~1.5 mm in the presence of an external acoustic field. However, there is a noticeable difference in the spatial distribution of the ions along the x-axis. While the *m/z* 37 ions decreased as they travelled further away from the source exit, the abundance of *m/z* 32 ions slightly increased along the downstream travel path; this suggests that the O_2^+ ions were produced along the path of the ion beam. This observation implies that the precursors of O_2^+ ions are likely ionic species, seeing as if they were produced by neutral species and later deflected towards adjacent nodes, they would have been detected inside the

antinode as well. In future studies, ion production under acoustic fields will be explored in a more detailed fashion.

Cross validation of ion deflection with an ionCCD instead of a mass spectrometer

In addition to using mass spectrometers for ion detection, a linear ion-sensitive charge-coupled device (ionCCD) was utilized to capture the one-dimensional profile of the total ion distribution (Fig. S5a). Unlike mass spectrometers, the ions in this setup directly reach the detector surface without passing through an atmospheric-pressure interface or sampling capillary. This direct view allows for a more straightforward observation of the acoustic-induced ion deflection.

The similarity between the observations of a mass spectrometer and the ionCCD indicates that all ions were deflected from an antinode to its adjacent node. The ions were then measured by the detector, which showed the deflection of the ion trajectory (Fig. S5b). Since plasma-based ionization sources can produce ions with both polarities, it was possible to detect negative ions by switching the ionCCD mode to negative mode and applying a bias voltage on the source exit capillary. Importantly, the negative ions exhibited the same deflection direction and pattern as the positive ions (Fig. 5c). Namely, all ions were deflected towards the node together.

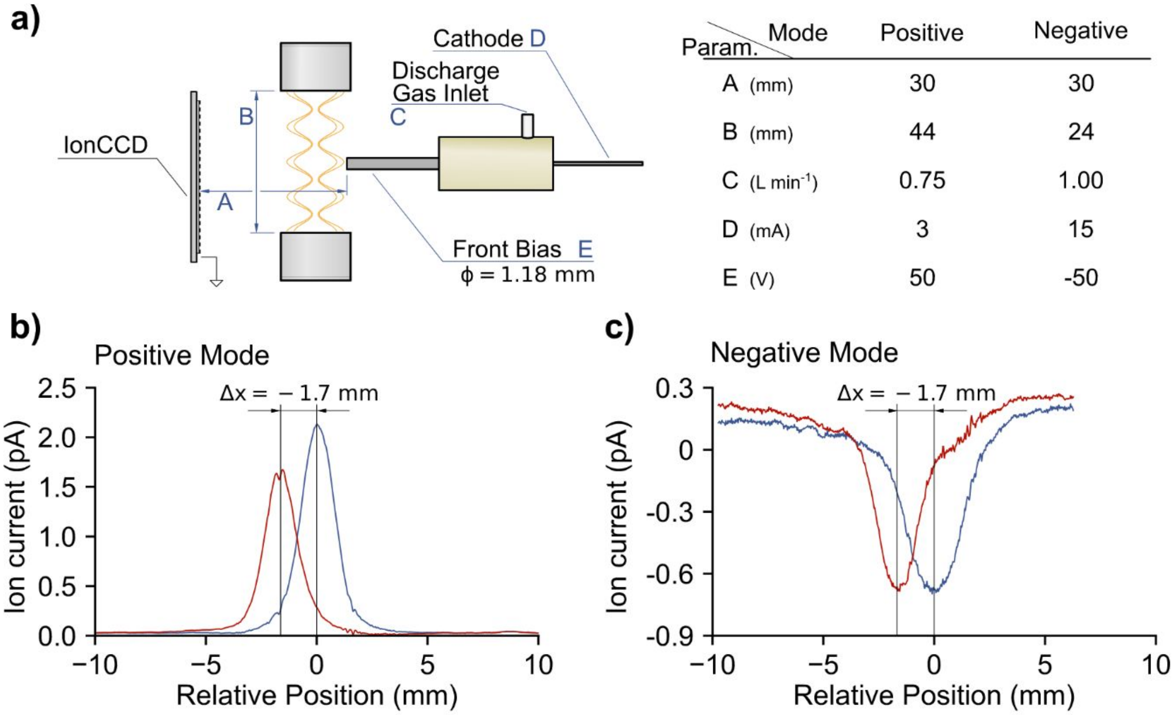

Figure S5. Cross validation of ion deflection with an ionCCD. The schematic and dimensions are given in panel (a). The voltage applied to the transducers was 20 $V_{p\text{-}p}$. Panel (b) and (c) represent the ion deflection of positive and negative ions, respectively.

3. Optical characterization of the linear acoustic resonator

A variant of a phase-sensitive technique termed defocusing (DF) shadowgraphy was used to visualize the transient acoustic field and the neutral gas stream, which is described in detail elsewhere (*3*). Briefly, the setup for DF shadowgraphy is similar to a co-linear schlieren imaging system, with the exception that the razor blade at the first focal point or Fraunhofer plane is not used. Instead, the objective in front of the imaging sensor is intentionally defocused by distance đ, acting as a retarder that makes phase distortions visible through observable fringes.

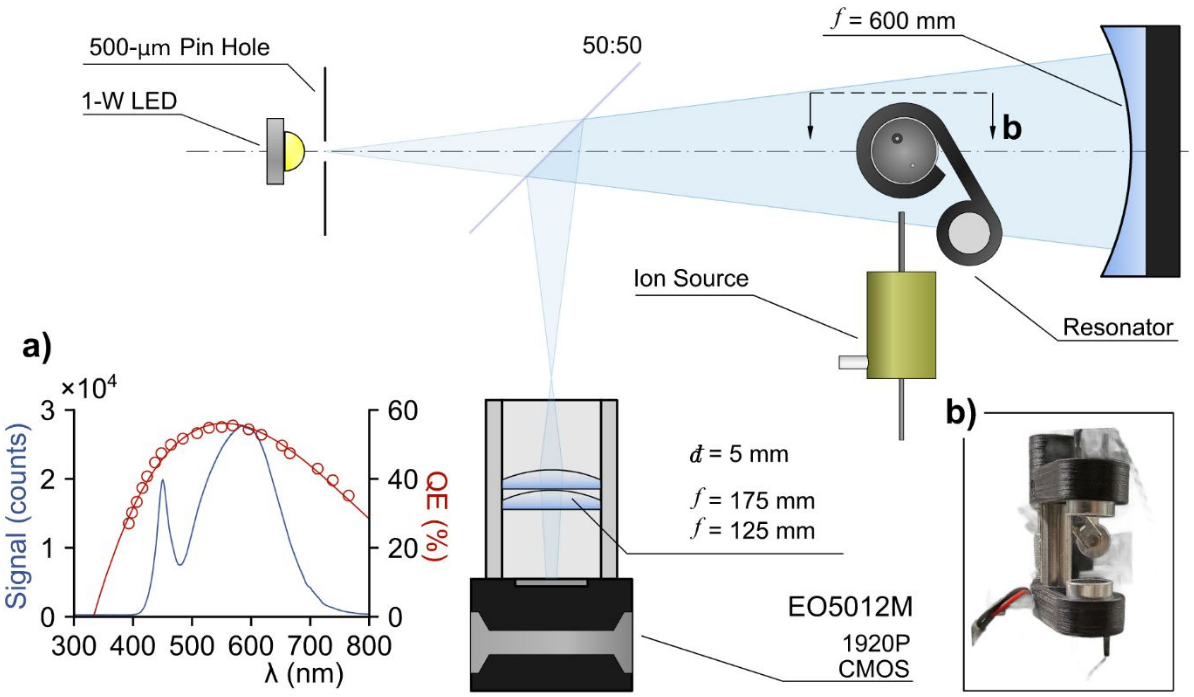

Figure S6. Schematic of the setup of the defocusing (DF) shadowgraphy system. The distance between the lens and the image focusing position is denoted by the symbol "đ". The emission spectrum and the quantum efficiency of the image detector (EO5012M, Edmund Optics) are shown in (a) with blue and red traces, respectively. In (b), a sectional view of the assembly housing the acoustic resonator and the ion source is provided. The outer diameter of the transducer is 16 mm, serving as a reference.

In this section of optical characterization, we utilized a function generator (DG1062Z, 60 MHz, 20 V_{p-p}, Rigol, Beijing, China) to power the ultrasonic transducers instead of using a home-built power supply. Notably, a sinusoidal waveform was used to drive the resonator, which is different from the driving circuit shown in the previous section (Fig. S3a). The sine wave frequency was set to 40 kHz. Additionally, the second channel of the function generator was utilized to generate a pulse-width modulation signal at 39.9996 kHz, with a pulse width of 3 µs, allowing for stroboscopic acquisition on the camera. The camera was operated at a frame rate of ~4 frames per second (2560x1920 resolution) using the internal trigger.

After a 60-second sampling period, an image representing the average over time can be obtained (Fig. 7a). Throughout this period, the acoustic field remained active. The data cube (x, y, t) was Fourier transformed only in the time domain, yielding energy spectra that typically look like that in Fig 7c. Due

to the high signal-to-background ratio in this scenario, a box bandpass filter is sufficient to isolate the time-dependent and time-independent components. By applying a bandpass filter of 0.4±0.1 Hz, the real and imaginary parts of the image can be obtained, which reflect the acoustic field (Fig. 7de).

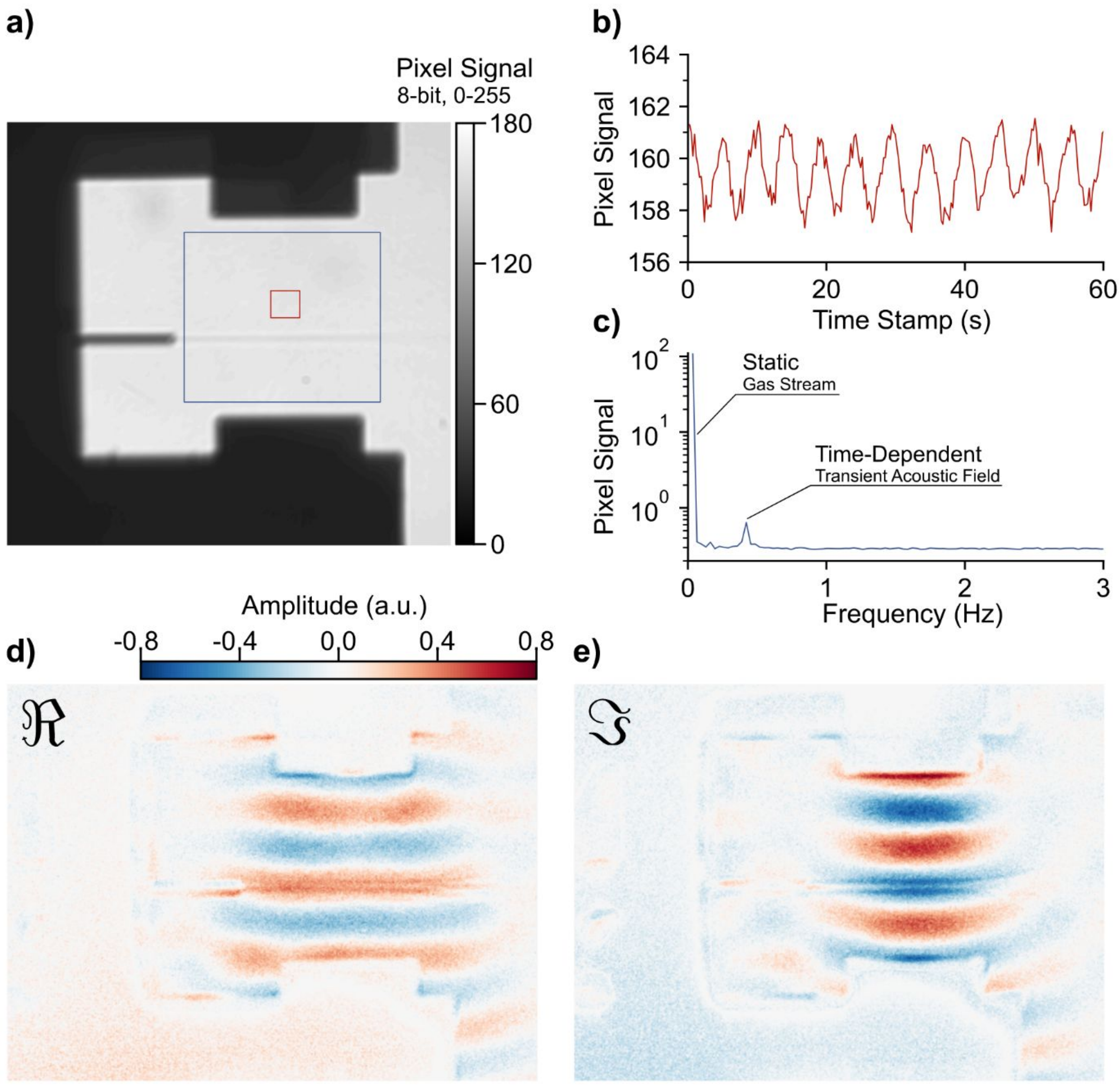

Figure S7. Extraction of the resonant acoustic field through energy spectrum analysis. The averaged camera image is shown in (a). Panel (b) shows the time-dependent averaged pixel signal in the red box in (a). Panel (c) presents the energy spectrum after Fourier transform of the averaged pixel signal in the blue box in (a). Panels (d) and (e) illustrate the real and imaginary parts of the image with a band-pass filter at 0.4 Hz.

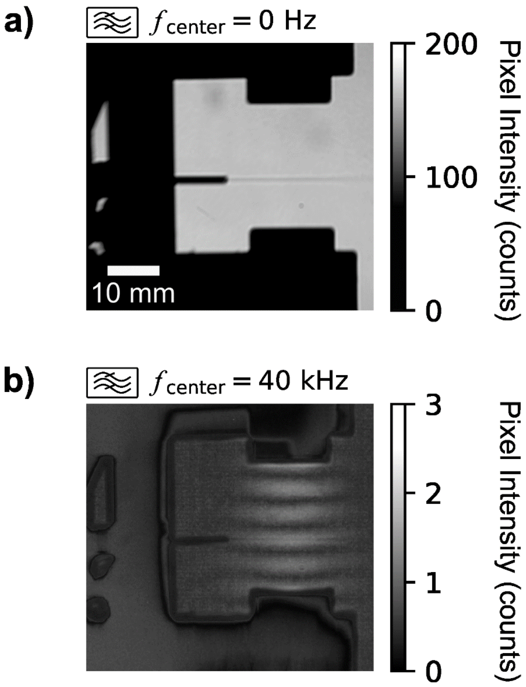

Fig. S8. Direct observation using the defocusing shadowgraphy method of a neutral gas stream containing a trace amount of isopropanol travelling through an antinode (bright regions in b) within the acoustic resonator. The gas velocity and the voltage applied to the transducer were 0.85 L min^{-1} and 30 $V_{p\text{-}p}$, respectively, which are the same condition given in Fig. 2 in the main text and as in the ion deflection experiment. Note that no electrical plasma was present, so the gas stream from the source is entirely neutral.

4. AIM ion cluster fractionation

In the same experiment, or in-line configuration of operation, we also investigated the acoustic-ion response curves associated with protonated water and ammonia clusters, as shown in Fig. S9. Interestingly, when the ion species related to ammonia and water were plotted together (Fig. S9), they exhibited highly similar acoustic-ion response curves, which can be accurately described by a single sigmoidal function (Fig. S9, represented by the black dashed curve).

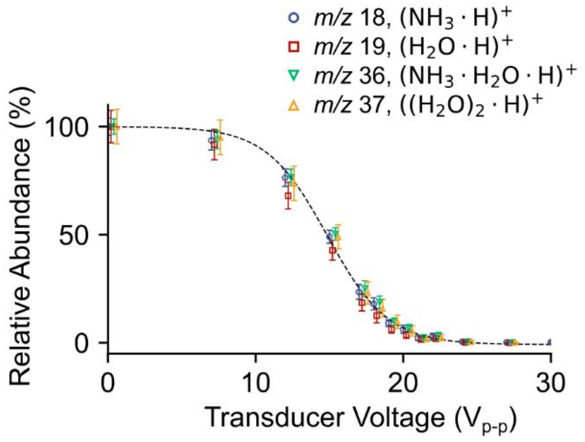

Figure S8. Acoustic-ion response curve for ion species closely related to water/ammonia. The markers of the four selected species are intentionally shifted horizontally for clarity.

In addition to the in-line configuration, or gating mode of operation, we introduce another configuration called deflection mode. In this mode, the inlet of the mass spectrometer was shifted vertically by half of the resonator wavelength of the acoustic standing wave (i.e., ~2.23 mm, as shown in Fig. S10a). The goal was for the ions to travel along the extension line of the ion outlet capillary and end up on top of the mass spectrometer inlet. Without the presence of the resonant acoustic field, the ions would not be detected. As the strength of the acoustic field increased, ions started to be detected (Fig. S10b) until a certain transducer voltage of ~30 $V_{p\text{-}p}$. However, further increasing the acoustic field strength caused a blockage effect. At this point, the ions could no longer pass through the acoustic field due to the high field strength. To analyze the response curves for different ions, a kernel that contains the sum of two sigmoidal functions was used. One sigmoidal function represented the ion deflection towards the ion detector, while the other sigmoidal function represented the blocking of ions from entering the resonance acoustic field. The curve fitting indicated a critical transducer voltage at ~27.5 $V_{p\text{-}p}$.

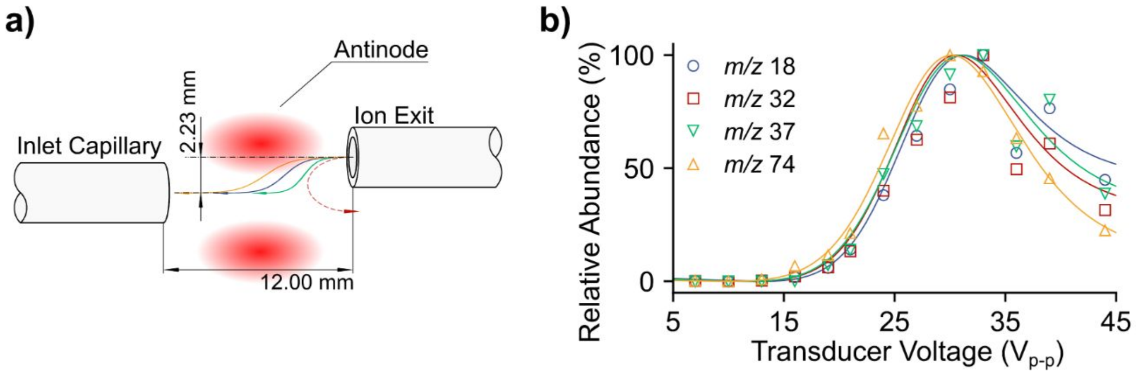

Figure S9. Deflection mode operation of AIM. The schematic of off-axis configuration is given in (a). The response curves for $NH_3 \cdot H^+$, O_2^+, $(H_2O)_2 \cdot H^+$ and $(CH_3OH \cdot CH_3CN \cdot H)^+$ at m/z 18, 32, 37, and 74, respectively, are shown in (b). The recorded values are represented by markers, while the fitting curves with a double sigmoidal kernel are shown in the same color.

5. Appendix

Front view of the two-dimensional acoustic resonator configuration

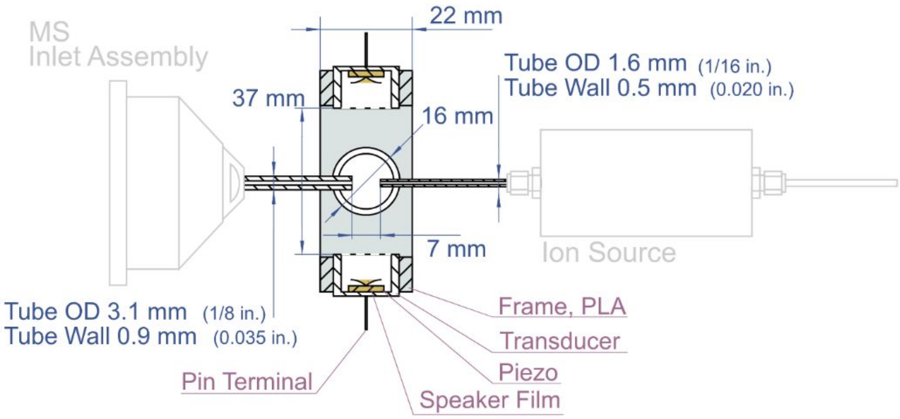

6.

Figure S10. Dimensional drawing of the two-dimensional acoustic resoantor and its relative positions to the mass spectrometer inlet capillary and the ion source exit. The parts represent in light gray indicated non-sliced view.

OpenCL kernel code for image rendering

All three-dimensional raster-scanned data were processed with OpenCL 3.0, including bootstrapping and rendering. For processing, an accelerated processing unit (APU, AMD Ryzen 7 7735HS, Advanced Micro Devices, CA).

Code, C-99

```
__kernel void bootstrap_3D(
    __global float4 *data,
    __global float3 *randomPositions,
    const int num_data,
    __global float *R,
    __global float *result
    )
{
    int id = get_global_id(0);

    float value_sum = 0.0f;
    int count = 0;

    for (int i = 0; i < num_data; i++) {
        float R1 = (data[i].x - randomPositions[id].x) * (data[i].x - randomPositions[id].x) +
             (data[i].y - randomPositions[id].y) * (data[i].y - randomPositions[id].y) +
             (data[i].z - randomPositions[id].z) * (data[i].z - randomPositions[id].z);
        if (R1 < R[id] * R[id])
        {
```

```
            value_sum += data[i].w;
            count++;
        }
    }

    if (count > 0)
    {
        result[id] = value_sum / count;
    }
    else
    {
        result[id] = 0.0f;
    }
}
```

Code, C-99

```
__kernel void rasterize(
       __readonly float3* data,
       const float xStep,
       const float yStep,
       const float xMin,
       const float yMin,
       const int width,
       const int height,
       __global volatile uint* pixelData
       )
{
    int id = get_global_id(0);
    int xIndex = (int)((data[id].x - xMin) / xStep);
    int yIndex = (int)((data[id].y - yMin) / yStep);

    int index = yIndex * width + xIndex;

    uint val = (uint)data[id].z;

    if (index < width * height)
    {
        atomic_max(&pixelData[index], val);
    }
}
```

7. References

1. J. T. Shelley, J. S. Wiley, G. M. Hieftje, Ultrasensitive ambient mass spectrometric analysis with a pin-to-capillary flowing atmospheric-pressure afterglow source. *Analytical chemistry* **83**, 5741-5748 (2011).
2. J. Cobos, O. Garcia, J. Sebastian, J. Uceda, in *Proceedings of 1994 IEEE Applied Power Electronics Conference and Exposition-ASPEC'94*. (IEEE, 1994), pp. 703-708.
3. Y. You, J. Riedel, Approaching phase-imaging through defocusing shadowgraphy for acoustic resonator diagnosis and the capability of direct index-of-refraction measurements. *Review of Scientific Instruments* **92**, (2021).
